# Is there a connection between sublingual varices and hypertension?

**DOI:** 10.1186/s12903-015-0054-2

**Published:** 2015-07-11

**Authors:** Lennart Hedström, Margit Albrektsson, Håkan Bergh

**Affiliations:** Public Dental Services, Box 1254, S-43218 Varberg, Sweden; Research & Development Unit, County of Halland, 30185 Halmstad, Sweden

**Keywords:** Sublingual varices, Hypertension, Blood pressure, Preventive health care

## Abstract

**Background:**

Sublingual varices have earlier been related to ageing, smoking and cardiovascular disease. The aim of this study was to investigate whether sublingual varices are related to presence of hypertension.

**Methods:**

In an observational clinical study among 431 dental patients tongue status and blood pressure were documented. Digital photographs of the lateral borders of the tongue for grading of sublingual varices were taken, and blood pressure was measured. Those patients without previous diagnosis of hypertension and with a noted blood pressure ≥ 140 mmHg and/or ≥ 90 mmHg at the dental clinic performed complementary home blood pressure during one week. Those with an average home blood pressure ≥135 mmHg and/or ≥85 mmHg were referred to the primary health care centre, where three office blood pressure measurements were taken with one week intervals. Two independent blinded observers studied the photographs of the tongues. Each photograph was graded as none/few (grade 0) or medium/severe (grade 1) presence of sublingual varices. Pearson’s Chi-square test, Student’s t-test, and multiple regression analysis were applied. Power calculation stipulated a study population of 323 patients.

**Results:**

An association between sublingual varices and hypertension was found (OR = 2.25, *p* < 0.002). Mean systolic blood pressure was 123 and 132 mmHg in patients with grade 0 and grade 1 sublingual varices, respectively (p < 0.0001, CI 95 %). Mean diastolic blood pressure was 80 and 83 mmHg in patients with grade 0 and grade 1 sublingual varices, respectively (p < 0.005, CI 95 %). Sublingual varices indicate hypertension with a positive predictive value of 0.5 and a negative predictive value of 0.80.

**Conclusions:**

An association was found between sublingual varices and hypertension. Examining the lateral borders of the tongue is easily done, causes no harm and could be a valuable method for the dental profession to take active part in preventive healthcare.

## Background

Hypertension is the primary risk factor for global disease burden [1] and causes organ damage already in the pre-symptomatic phase, before causing cardiovascular disease such as stroke and myocardial infarction. It is therefore important to discover and treat hypertension as early as possible. In Sweden, there is today no widespread structured screening for hypertension among healthy individuals. However, blood pressure is generally acquired when people are seeking health care for other reasons.

A major part of the population visits their dentist on a yearly basis. Approximately 80 % of Swedish grown-ups are in contact with a dental team by a recall system, and about 60 % visit the dental team yearly. Together with a long tradition of prophylactic work, the dental team could potentially be a good screening partner for hypertension by investigating the patient’s oral cavity.

A common condition that may be found by the dentist is varicosities, a benign venous lesion. This condition can be found in several locations within the oral cavity; in the buccal mucosa, in the lower lip mucosa, and most often under the lateral borders of the tongue, i.e. sublingual varices [2].

Sublingual varices are clinically characterised by small dilated veins under the lateral borders of the tongue. Their pathogenesis may be due to a change in the connective tissue or weakening of the venous wall, as a result of degeneration of elastic fibres related to the ageing process [3–5]. The phenomenon of sublingual varices is not well studied and it may to be correlated with age, smoking and cardiovascular disease and varicose veins of the leg [6–9]. A connection between sublingual varices and hypertension has not yet been shown.

The aim of this study was therefore to investigate whether sublingual varices are related to presence of hypertension.

## Method

### Design and setting

This observational study was performed between May 2010 and February 2013 at the Public Dental Services, Varberg, Sweden.

### Study population

Patients above the age of 40 years were invited to participate in the study in connection to their regular yearly visit. They received both verbal and written information about the study. Those who accepted to participate provided written consent. Exclusion criteria were pregnancy, atrial fibrillation and renal disease [10]. The study was approved by the Regional Research Ethics Committee at the University of Lund (EPN 2009/204), and was performed in accordance with the Helsinki Declaration.

### Procedure

Patients answered a questionnaire about background information (age,sex) and health status (actual smoking, hypertension, atrial fibrillation, ischemic heart disease, myocardial infarction, stroke, other cardiovascular diseases, lower limb varices). One intraoral digital photograph of each lateral borders of the tongue were acquired before the routine oral survey. Thereafter, the patient rested for at least 5 min in a quiet room. Blood pressure was measured in a sitting position, 2 times in each upper arm using the Korotkoff-Riva-Rocci method, using a cuff, a calibrated manometer and a stethoscope. If a patient had no previous diagnosis of hypertension and the measured average systolic blood pressure was ≥ 140 mm Hg, and/or the average diastolic blood pressure was ≥ 90 mm Hg, the patient was asked to use a home blood pressure device (Omron M6 Comfort, Omron Healthcare Ltd, Kyoto, Japan) for 1 week [11]. The follow-up blood pressure measurements were acquired in the arm where the highest average blood pressure was noted. If the average blood pressure was equal between arms, the patient decided on his own which arm to apply follow-up measurements. The patient was given both verbal and written instructions on how to use the home blood pressure device [12]. The home blood pressure was measured twice daily, at morning and evening time, with 2 measurements acquired at each time point. The time of acquisition and blood pressure were noted on an enclosed form. The form and home blood pressure monitoring device were returned to the study team for calculating the average blood pressure from day 2–7 [12].

If the average home blood pressure was systolic ≥135 and/or diastolic ≥85 mm Hg the patient was referred to his ordinary primary health care centre [10, 12], where 3 office blood pressure measurements were acquired 1 week apart [13]. If the average office blood pressure was systolic ≥140 and/or diastolic ≥90 mm Hg the patient was diagnosed with hypertension, and the result was reported back to the study team. An average systolic blood pressure ranging from 140 to 159 mm Hg and or a diastolic pressure ranging from 90 to 99 mm Hg was classified as stage 1 hypertension, and an average systolic pressure of 160 mm Hg or higher and or a diastolic pressure of 100 mm Hg or higher as stage 2.

**Fig. 1:**
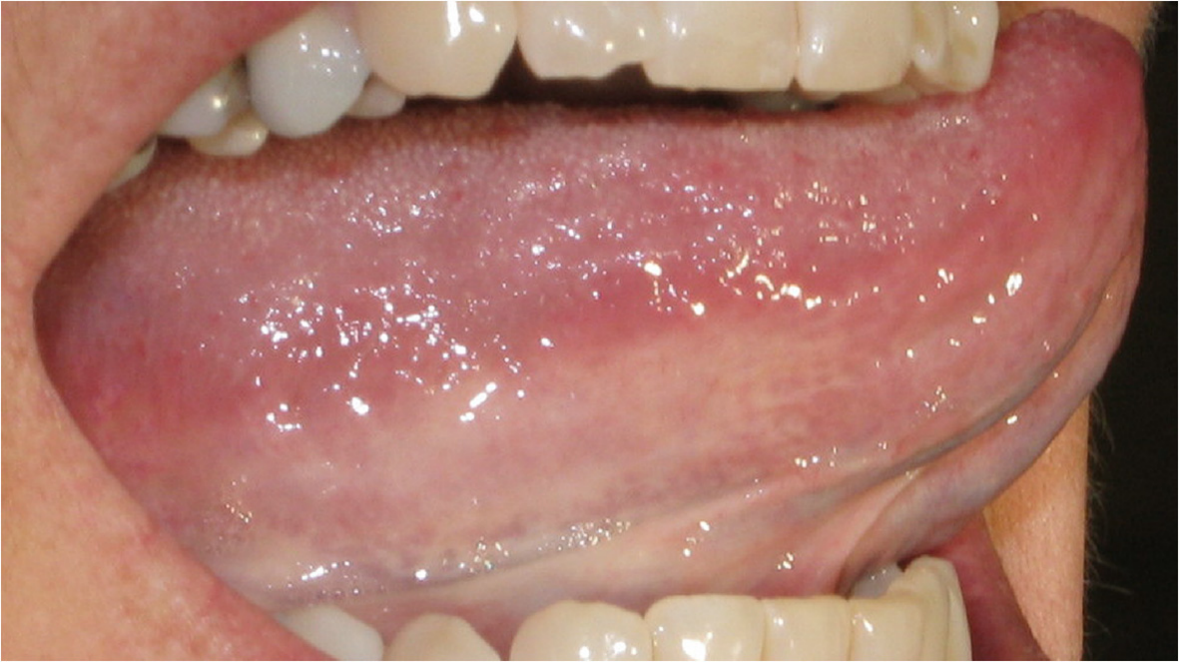
None/few sublingual varices (grade 0). Written consent obtained from patient

**Fig. 2:**
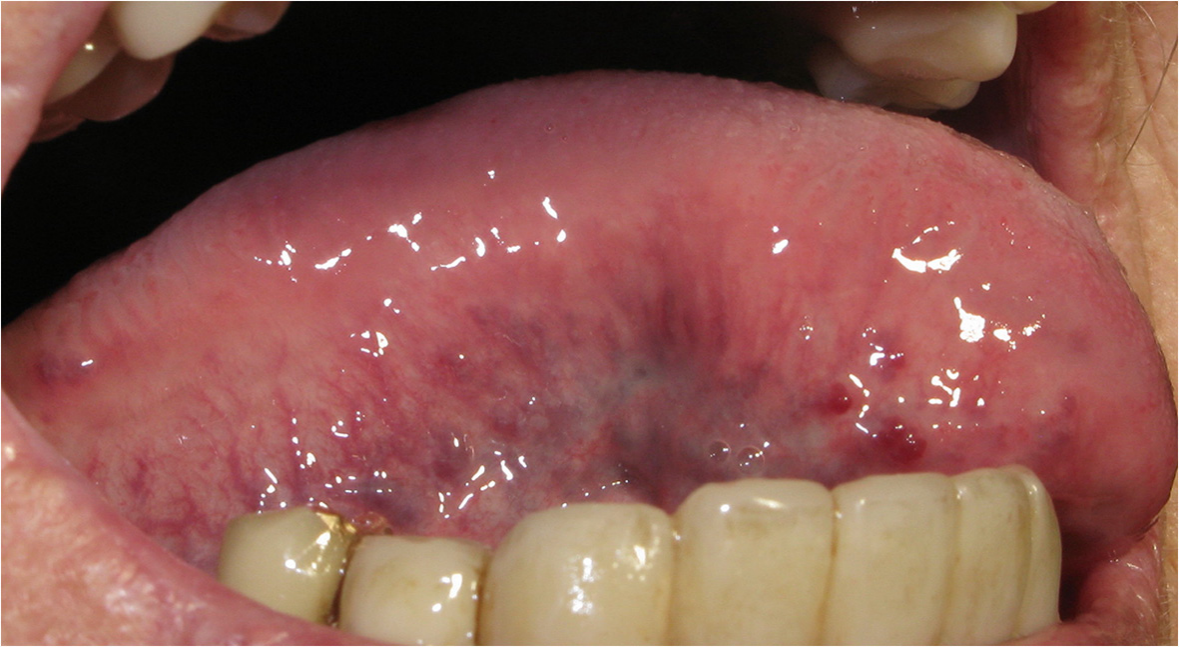
Moderate/severe sublingual varices (grade 1). Written consent obtained from patient

The two digital photographs of each patient were examined in a blinded fashion by 2 observers (one of the authors and one dentist not involved in the study) not having knowledge about the other’s results nor the patient’s blood pressure or health status. Both observers viewed the photographs using the same screen. All photographs were graded as none/few visible sublingual varices (grade 0; Fig. 1) or medium/severe sublingual varices (grade 1; Fig. 2). Consensus was reached in cases where the initial assessment differed between observers.

### Statistical analysis

The variables from the questionnaire (sex, actual smoking, hypertension, atrial fibrillation, ischemic heart diseases, myocardial infarction, stroke, other cardiovascular diseases, lower limb varices) were coded as dummy variables. All statistical analyses were performed using IBM SPSS Statistics version 20. Pearson’s Chi-square test, Student’s t-test, and multiple logistic regression analysis were applied. Correlation between observers was analysed using Cohen’s kappa coefficient. Descriptive statistics with index of validity, sensitivity and specificity were also used. All tests were two-sided and the significance cut-off was set at 0.05.

The expected prevalence of hypertensive patients in a Swedish population above 40 years of age is 38 % [14]. The prevalence of sublingual varices in patients with cardiovascular disease is approximately 70 %, compared with 27 % for patients without cardiovascular disease [9]. Power calculations using these data as input resulted in a sample size of 323 patients, considering an alpha error of 0.05 and a beta error of 0.20.

## Results

**Fig. 3:**
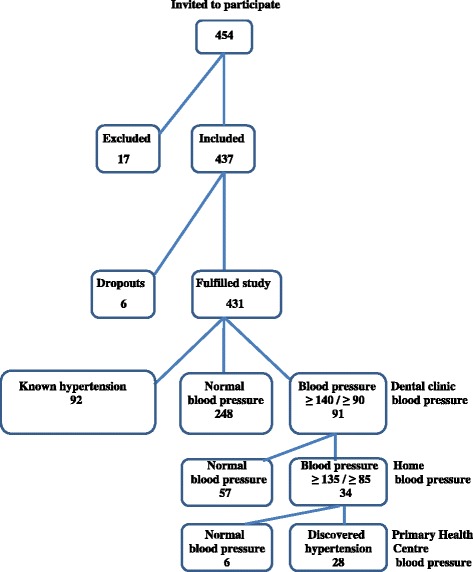
Flowchart of the study population

A total number of 454 patients were invited to participate in the study. Seventeen patients

(3.7 %) resigned from the study (fifteen patients did not want to enter the study, two patients were excluded due to atrial fibrillation). Of the remaining 437 patients, 2 were excluded due to lack of photographs of the tongue, and 4 patients refused to visit the district nurse for follow-up after initial high blood pressure was observed at inclusion. In total 431 patients underwent the complete study protocol. The total dropout number was thus 23 patients (5.1 %; Fig. 3).

The 431 patients who underwent the complete study protocol had a mean age of 55.3 years (SD 10.9). The gender distribution was 188 male and 243 female. The prevalence of smokers was 46 (10.7 %), and 202 (46.9 %) expressed heredity for hypertension. For 92 patients, previously known hypertension was reported (21.3 %) with a mean duration from diagnosis of 7.2 years (SD 7.2), and another 28 patients (6.5 %) had other cardiovascular disease (angina pectoris, myocardial infarction or stroke) and 30 patients (7.0 %) had lower limb varices. During the study another 28 participants were identified having hypertension (Fig. 3). A total of 120 (27.8 %) individuals were thereby hypertensive in the current study.

The prevalence of sublingual varices was 26.5 % (114/431) in the study population, with 18.3 % among those having normal blood pressure, and 47.5 % among those diagnosed with hypertension (*p* < 0.0001).

**Fig. 4:**
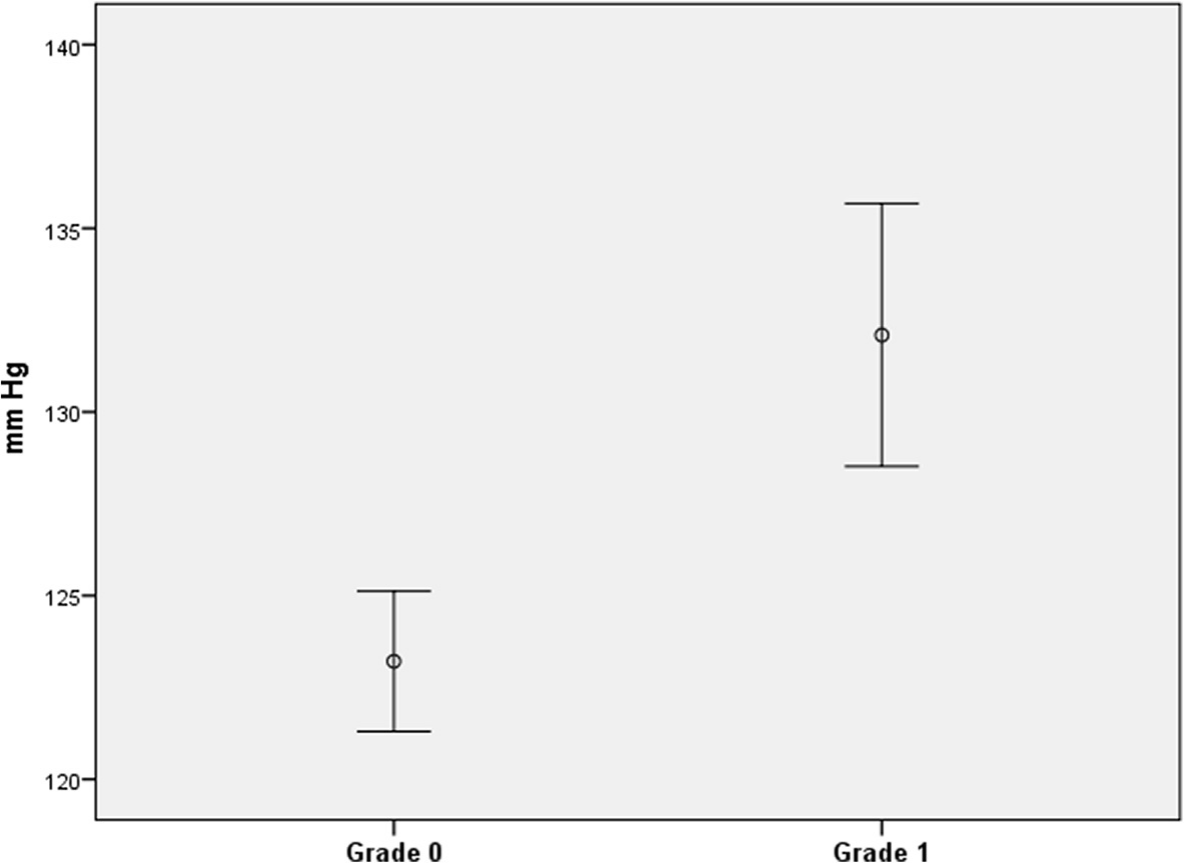
Mean systolic blood pressure with 95 % confidence intervals in patients with grade 0 (*n* = 317) and grade 1 (*n* = 114) sublingual varices, respectively

**Fig. 5:**
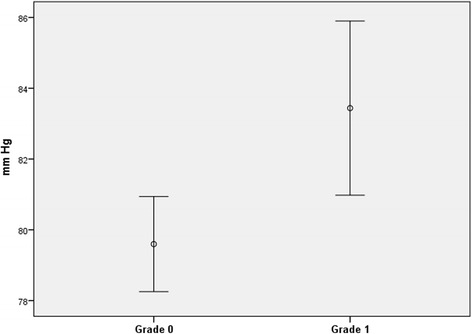
Mean diastolic blood pressure with 95 % confidence intervals in patients with grade 0 (*n* = 317) and grade 1 (*n* = 114) sublingual varices, respectively

The mean systolic blood pressure measured at inclusion at the dental clinic was 123.2 mm Hg (SD 17.3) in the group with grade 0 sublingual varices and 132.1 mm Hg (SD 19.3) in the group with grade 1 sublingual varices (Fig. 4; p < 0.0001). The mean diastolic blood pressure was 79.6 mm Hg (SD 12.2) in the group with few or no sublingual varices (grade 0) and 83.4 mm Hg (SD 13.3) in the grade 1 sublingual varices group (Fig. 5; *p* = 0.005).

The prevalence of grade 1 sublingual varices increased with increasing blood pressure measured at inclusion at the dental clinic, from 21.8 % among those with normal blood pressure to 30.8 % among those with stage 1 hypertension (*p* = 0.02), and further to 43.6 % among those with stage 2 hypertension (*p* = 0.001).

In the group with newly diagnosed hypertension at the primary health care centre (*n* = 28), the prevalence of grade 1 sublingual varices was 46.2 % and 53.3 % in patients with stage 1 hypertension and stage 2 hypertension , respectively.

Age, smoking and hypertension were associated with sublingual varices (Table 1). The prevalence of sublingual varices among non-smokers with hypertension was 44.7 % (46/103), and among smokers with hypertension 64.7 % (11/17).

**Table 1:** Sublingual varices as the dependent variable (odds ratio (OR), p-value and 95 % confidence interval (CI)) by logistic regression (*n* = 431), adjusted for cardiovascular disease and lower limb varices

Variable	OR	*p*- value	95 % CI
Age	1.07	<0.0001	1.04 – 1.09
Smoking	2.22	0.025	1.11 – 4.45
Hypertension	2.25	0.002	1.34 – 3.77

The identification of sublingual varices as an indicator of hypertension would result in a sensitivity of 0.48, a specificity of 0.82, a positive predictive value (PPV) of 0.5, and a negative predictive value (NPV) of 0.80 (Table 2). Sublingual varices as an indicator of hypertension in the subgroup of smokers showed a sensitivity of 0.65, a specificity of 0.2, a PPV of 0.58, and an NPV of 0.25.

**Table 2:** Distribution of sublingual varices in hypertensive and normotensive patients (*n* = 431)

	Hypertensive	Normotensive
	n (%)	n (%)
Sublingual varices grade 0	63 (19.9)	254 (80.1)
Sublingual varices grade 1	57 (50.0)	57 (50.0)
	120	311

The inter-observer agreement regarding photographs of the tongue, expressed as index of validity, was 0.87 and the Cohen’s kappa coefficient was 0.68.

## Discussion

An association between sublingual varices and smoking, age and hypertension was found. The prevalence of sublingual varices increased with stage of hypertension, further confirming the connection to increased blood pressure. The correlation between sublingual varices and hypertension has not been previously shown.

Investigations regarding sublingual varices have earlier been related to histopathology [3, 4], pathogenesis [5], and to the relation between sublingual varices and cardiovascular disease [6, 7]. In a survey from 1968 Bhaskar et al. [15] could not reveal a correlation between sublingual varices and hypertension, based on 384 cases. In a retrospective study including 281 adults, a strong correlation was found between sublingual varices and cardiovascular disease. The majority of patients with cardiovascular disease in that study had a diagnosis of hypertension (74 %). The diagnosis of hypertension was however self-reported and not verified by blood pressure measured [9].

In the current study the prevalence of sublingual varices was higher in patients with hypertension diagnosed at the primary health care centre compared to those classified as hypertensive at the dental clinic. This difference may in part be explained by falsely high blood pressure acquired at the dental clinic due to the white-coat effect, as these patients were found to be normotensive by home blood pressure. Possible explanations to the connection between sublingual varices and hypertension could include circulatory anastomosis in the venous system of the tongue, though there are divergent opinions in the literature [2], or a hemodynamic effect where the arterial pressure influences the veins through arteriovenous shunts [16].

The low drop-out frequency of 5.1 % strengthens the results. A potential weakness of the study lies in estimating whether a patient has sublingual varices. To minimise this potential issue, standardised digital photographs were acquired. The high inter-observer agreement also indicates the effect of this standardisation [17].

A clinical implication of this study is the possibility to use sublingual varices as an indicator of risk for hypertension. Examining the lateral borders of the tongue in at the yearly dental visit is easily and quickly performed, and causes no harm for the patient. Screening for hypertension using identification of sublingual varices (grade 1) in the current study population would identify 48 % of hypertensive patients, with a high specificity of 0.82.

Based on the results of the current study, a patient above 40 years of age with sublingual varices has a 50 % risk of being hypertensive, and if also being a smoker the risk increases to 58 %. If sublingual varices are not present the chance of being normotensive is 80 %. The high positive predictive value for hypertension, combined with no discomfort for the patient and minor effort for the dentist, makes sublingual varices highly interesting as a clinical sign to note in the oral status.

## Conclusion

An association was found between sublingual varices and hypertension in patients over 40 years of age. With a sensitivity of 0.48, a specificity of 0.82, a PPV of 0.5, and an NPV of 0.80 the identification of sublingual varices is highly interesting as an indicator of hypertension. Examining the lateral borders of the tongue is easily carried out by a dentist, causes no harm to the patient, and could be a valuable method for the dental profession to take active part in preventive healthcare.
